# Treatment time metrics and functional outcomes after endovascular thrombectomy in routine clinical practice

**DOI:** 10.3389/fneur.2026.1808012

**Published:** 2026-08-21

**Authors:** Yu Qiao, Yan Yan, Tengfei Guo, Liyun Wang, Lin Wang

**Affiliations:** 1 Department of Neurology IV, Xingtai People’s Hospital, Xingtai, Hebei, China; 2 Department of Neurology VI, Xingtai People’s Hospital, Xingtai, Hebei, China; 3 Department of Neurology I, Longyao County Hospital, Xingtai, Hebei, China

**Keywords:** acute ischaemic stroke, endovascular thrombectomy (EVT), endovascular treatment, modified Rankin scale outcomes, safety

## Abstract

**Background:**

Randomised clinical trials have shown that functional outcomes after acute ischaemic stroke decline with each hour of delay to endovascular treatment. However, these estimates may not fully reflect real world practice because trial populations are highly selected and workflow conditions differ from routine care. We therefore examined the associations of prehospital and in-hospital treatment times with functional outcomes and safety endpoints among patients undergoing endovascular thrombectomyat a large comprehensive stroke centre.

**Methods:**

We conducted a study of consecutive patients with large vessel occlusion treated with endovascular therapy between 2024 and 2025. The primary outcome was the modified Rankin Scale at 90 days, analysed using ordinal logistic regression. Secondary outcomes included excellent, independent, and favourable recovery, mortality, successful reperfusion, and symptomatic intracranial haemorrhage. Models were adjusted for demographic factors, prestroke functional status, baseline stroke severity, infarct burden, and occlusion characteristics.

**Results:**

Among 288 patients, longer onset-to-puncture time was independently associated with worse functional outcome. Compared with treatment achieved within less than 4.5 h, onset-to-puncture intervals of 4.5 to 6 h and greater than 6 h were associated with higher odds of poorer modified Rankin Scale outcomes. Delays beyond 6 h were also associated with increased 90-day mortality. No significant associations were observed between treatment timing and successful reperfusion or symptomatic intracranial haemorrhage.

**Conclusion:**

In routine clinical practice, shorter onset-to-puncture times are associated with more favorable functional outcomes after endovascular thrombectomy, whereas treatment beyond 6 h is associated with higher mortality. These findings highlight the importance of minimizing avoidable delays in acute stroke care while underscoring the need to interpret time-based metrics within the context of patient selection and stroke heterogeneity.

## Introduction

Endovascular thrombectomy (EVT) is the standard of care for acute ischemic stroke (1, 2) caused by large vessel occlusion (LVO), offering substantial benefits in reducing disability and improving survival when performed rapidly after symptom onset. The benefit of EVT, however, is strongly time dependent (3–5), with shorter intervals from stroke onset to reperfusion associated with higher rates of successful reperfusion and better clinical outcomes. This principle has been repeatedly confirmed across both randomized trials and real-world observational studies, reinforcing the central role of treatment timeliness in acute stroke care (6, 7).

Despite growing recognition of the importance of minimizing delays, significant variation remains in EVT workflow performance across centers and health systems (8). While time from symptom onset to arterial puncture has been widely studied, it comprises multiple distinct intervals, including onset-to-arrival, arrival-to-puncture, and puncture-to-reperfusion (9), that may be influenced by different operational or clinical factors. The specific contribution of each time interval to patient outcomes, and whether they exert differential effects, remains incompletely understood (9). Moreover, most time-outcome analyses have focused on selected trial populations (8) or highly resourced centers, limiting generalizability to broader stroke populations. Timely access to EVT remains a significant challenge in many regions (10), due to disparities in healthcare infrastructure and resources.

A more comprehensive evaluation of patient-centred outcomes in relation to EVT treatment time may therefore provide important complementary insights. Understanding how delays in treatment affect not only physical disability but also broader aspects of health-related quality of life is particularly relevant in the context of ongoing efforts to optimise stroke systems of care. For example, delays between hospital arrival and arterial access (door-to-puncture time) may reflect modifiable in-hospital processes, whereas longer onset-to-arrival times may be less amenable to change in certain geographic contexts. Therefore, in this study, we aimed to (1) to examine functional outcomes across commonly used prehospital and in-hospital treatment-time windows among patients treated with endovascular thrombectomy, and (2) to explore whether these associations differed across functional and safety outcomes.

## Methods

### Study design and population

This observational cohort study was conducted in accordance with the Strengthening the Reporting of Observational Studies in Epidemiology (STROBE) guidelines. The study population included consecutive adults aged 18 years or older who presented with acute ischemic stroke due to large vessel occlusion and were treated with endovascular therapy in a single comprehensive stroke centre from Dec 2024 to July 2025. All patients underwent standardised clinical assessment, neuroimaging, and EVT decision-making as part of routine clinical practice. As this was a retrospective observational study, all consecutive eligible patients treated during the study period were included. No formal prospective sample size calculation was performed.

### Data collection

We collected baseline data of demographics (age and gender), medical history, Imaging data, laboratory values, disability, and stroke severity. Medical history encompassed hypertension, diabetes, atrial fibrillation, dyslipidaemia, chronic kidney disease, and prior stroke. The modified Rankin Scale (mRS) (11) was assessed via in-person evaluation with a neurologist. Stroke severity was measured using the National Institutes of Health Stroke Scale (NIHSS) (12), with total scores ranging from 0 to 42 and higher values indicating more severe neurological impairment.

ASPECTS measures (13) the number of infarct regions by dividing the hemisphere fed by the middle cerebral artery into 10 regions. The ASPECTS value ranges from 0 to 10, with lower values indicating a greater infarct area. Imaging data included ASPECTS on non-contrast CT, infarct core volume on CT perfusion or MRI diffusion-weighted imaging, and occlusion site (internal carotid artery, M1 or M2 middle cerebral artery). ASPECTS measures the number of infarct regions by dividing the hemisphere fed by the middle cerebral artery into 10 regions. The ASPECTS value ranges from 0 to 10, with lower values indicating a greater infarct area. Laboratory biomarkers included baseline and 24-h levels of neuron-specific enolase (NSE), high-sensitivity C-reactive protein (hsCRP), and tumour necrosis factor-alpha (TNF-*α*), where available. Haemorheological parameters were also measured, including whole blood viscosity, plasma viscosity, and fibrinogen concentration, using an automated rheometer (LBY-N6C, Beijing Pulisheng Instrument Co., Ltd).

Two treatment-time metrics were collected: onset-to-puncture time, defined as the interval from last known well to arterial puncture, and arrival-to-puncture time, defined as the interval from hospital arrival to arterial puncture.

### Outcomes

The primary outcome was functional status at 90 days assessed using ordinal logistic regression of mRS, ranging from 0 (no symptoms) to 6 (death). Secondary outcomes included the proportions of patients achieving excellent outcomes (mRS score 0–1), functional independence (mRS score 0–2), and favourable outcomes (mRS score 0–3) at 90 days (14). An additional secondary outcome was early neurological improvement (ENI), defined as an NIHSS score of 0 or 1 at 36 h, or an improvement of at least 10 points from baseline at 36 h. Functional motor recovery at 90 days was measured using the Fugl-Meyer Assessment, evaluating motor function, balance, sensation, and joint movement following stroke, with higher scores indicating better motor recovery.

Safety outcomes included the incidence of symptomatic intracerebral haemorrhage (sICH) within 48 h, as defined by the Heidelberg Bleeding Classification, and all-cause mortality within 90 days.

### Statistical analysis

Patients were categorised into onset-to-puncture time groups to reflect clinically relevant treatment windows used in contemporary EVT practice. The onset-to-puncture interval was defined as the time from the last known well to arterial puncture. Based on guideline thresholds and prior EVT studies, we classified patients into three groups: less than 4.5 h, 4.5 to 6 h, and more than 6 h for the reference (15, 16). Patients were stratified according to the hospital arrival-to-arterial puncture time, with less than 60 min vs. 60 min or longer, consistent with recommended door-to-puncture benchmarks.

Descriptive statistics were used to summarise baseline characteristics. Outcomes were compared across onset-to-puncture and arrival-to-puncture categories using proportions with 95% confidence intervals. Multivariable regression analyses were performed to assess the association between treatment time, as a continuous and categorical variable, and outcomes. Ordinal logistic regression was used for the primary mRS shift outcome, and binary logistic regression was used for dichotomised functional, safety, and reperfusion outcomes (17). All models were adjusted for age, sex, prestroke mRS, baseline NIHSS, ASPECTS, ischemic core volume and occlusion site. Results are reported as odds ratios with 95% confidence intervals. Analyses were conducted using R (version 4.4.1).

## Results

In total, 288 patients with acute ischemic stroke treated with endovascular therapy were included in the analysis. The median (IQR) age was 69.0 (64.0–76.0) years, and 139 patients (48%) were male. Regarding smoking status, 139 patients (48%) were never smokers, 114 (40%) were former smokers, and 35 (12%) were current smokers. The median (IQR) baseline NIH Stroke Scale score was 17 (10–23), and the median (IQR) prestroke mRS score was 0 (0–1). For imaging characteristics, the occlusion site was the internal carotid artery in 65 patients (23%), the M1 segment of the middle cerebral artery in 170 (59%), and the M2 segment in 53 (18%). The median (IQR) Alberta Stroke Program Early CT Score (ASPECTS) was 6 (4–7), and the median (IQR) core infarct volume was 48.0 (38.0–62.5) mL. A total of 163 patients (57%) had a large infarct. Baseline characteristics are shown in Table 1.

**Table 1:** Baseline characteristics by onset-to-puncture time.

Variable	Overall (*N* = 288)	<4.5 h (*N* = 140)	4.5–6 h (*N* = 80)	>6 h (*N* = 68)
Age, years, median (IQR)	69.0 (64.0, 76.0)	70.0 (64.0, 76.0)	68.0 (63.0, 76.0)	70.0 (64.5, 76.5)
Sex, *n* (%)				
Female	149 (52%)	70 (50%)	41 (51%)	38 (56%)
Male	139 (48%)	70 (50%)	39 (49%)	30 (44%)
Smoking status, *n* (%)				
Current	35 (12%)	22 (16%)	5 (6.3%)	8 (12%)
Former	114 (40%)	53 (38%)	33 (41%)	28 (41%)
Never	139 (48%)	65 (46%)	42 (53%)	32 (47%)
Hypertension, *n* (%)	183 (64%)	89 (64%)	56 (70%)	38 (56%)
Diabetes, *n* (%)	95 (33%)	48 (34%)	26 (33%)	21 (31%)
Atrial fibrillation, *n* (%)	93 (32%)	56 (40%)	22 (28%)	15 (22%)
Dyslipidaemia, *n* (%)	154 (53%)	74 (53%)	40 (50%)	40 (59%)
Chronic kidney disease, *n* (%)	51 (18%)	23 (16%)	12 (15%)	16 (24%)
Prestroke modified Rankin Scale, median (IQR)				
0	178 (62%)	81 (58%)	55 (69%)	42 (62%)
1	61 (21%)	34 (24%)	12 (15%)	15 (22%)
2	31 (11%)	14 (10%)	11 (14%)	6 (8.8%)
3	13 (4.5%)	7 (5.0%)	1 (1.3%)	5 (7.4%)
4	5 (1.7%)	4 (2.9%)	1 (1.3%)	0 (0%)
Baseline NIHSS, median (IQR)	17.0 (10.0, 23.0)	15.0 (10.0, 22.0)	17.0 (11.5, 22.5)	19.0 (12.0, 23.0)
Baseline systolic BP, mmHg, median (IQR)	147.0 (132.5, 161.0)	146.0 (133.0, 159.0)	147.0 (128.5, 166.5)	150.5 (137.0, 164.0)
Baseline glucose, mmol/L, median (IQR)	8.3 (7.1, 9.7)	8.2 (6.9, 9.6)	8.4 (7.3, 9.6)	8.4 (7.2, 9.8)
ASPECTS, median (IQR)	6.0 (4.0, 7.0)	5.0 (4.0, 7.0)	6.0 (4.5, 7.0)	6.0 (5.0, 7.0)
Core infarct volume, mL, median (IQR)	48.0 (38.0, 62.5)	46.0 (35.5, 59.5)	49.0 (38.0, 64.5)	56.0 (42.5, 65.0)
Occlusion site, *n* (%)				
Internal carotid artery	65 (23%)	38 (27%)	19 (24%)	8 (12%)
M1 middle cerebral artery	170 (59%)	78 (56%)	45 (56%)	47 (69%)
M2 middle cerebral artery	53 (18%)	24 (17%)	16 (20%)	13 (19%)
Large infarct, *n* (%)	163 (57%)	84 (60%)	40 (50%)	39 (57%)

Median onset to puncture time was 199 min (IQR, 127 to 306 min), with a mean of 229.8 min and a range from 9 to 766 min. Median arrival to puncture time was 61 min (IQR, 47 to 73 min), and the mean value was 60.1 min, with a range from 10 to 119 min. 140 (48.6%) were treated within 4.5 h of symptom onset, 80 (27.8%) between 4.5 and 6 h, and 68 (23.6%) beyond 6 h.

**Figure 1:**
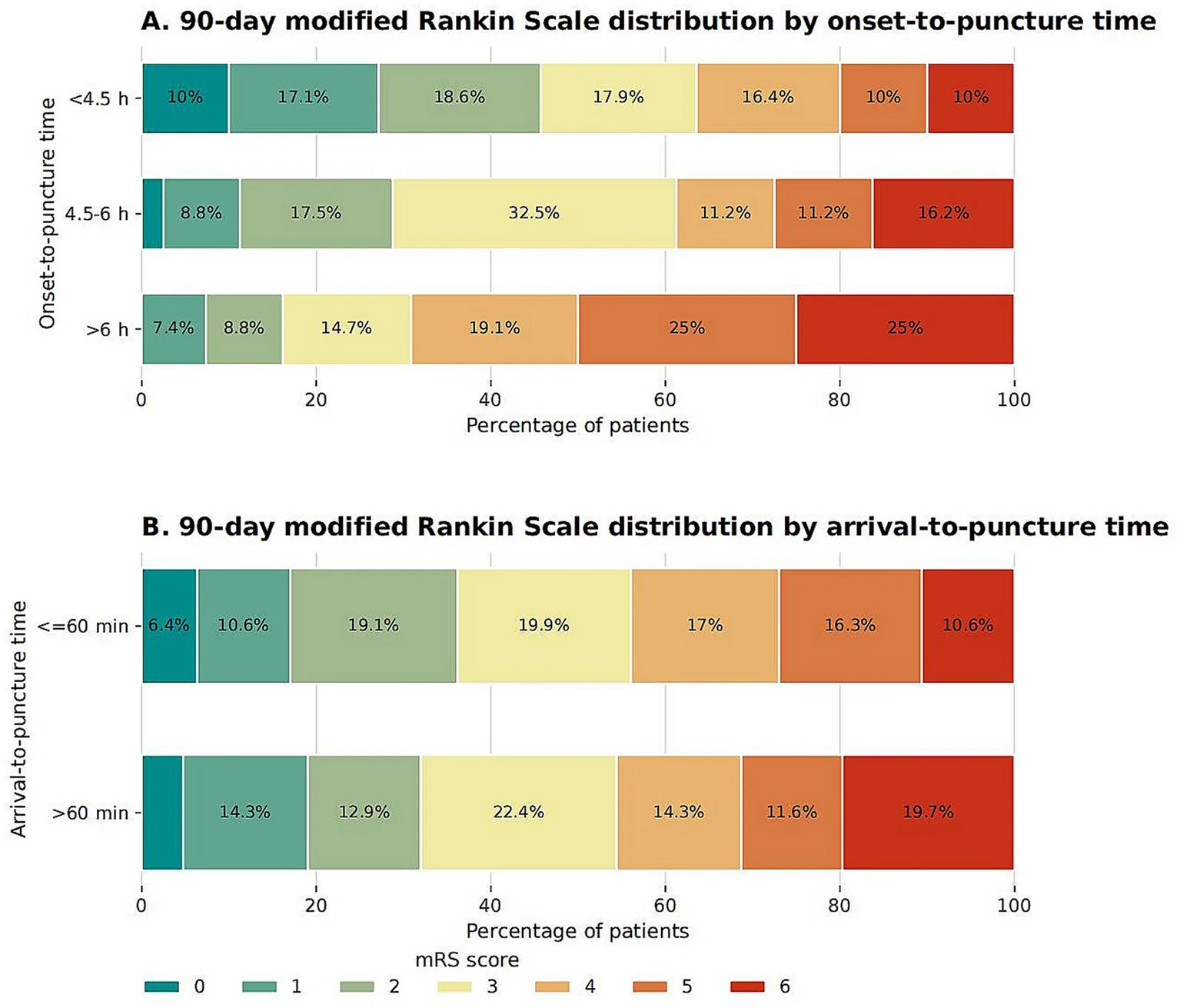
Distribution of 90-day mRS scores by treatment time. **(A)** Distribution of 90-day mRS scores by onset-to-puncture time; **(B)** Distribution of 90-day mRS scores by arrival-to-puncture time.

A total of 159 patients (55%) had a favourable outcome (mRS 0–3) at 90 days, with 52 patients (18%) achieving an excellent outcome (mRS 0–1), and 98 patients (34%) achieved functional independence (mRS 0–2). In patients treated within 4.5 h, a greater proportion achieved lower disability scores: 45.7% had mRS 0–2, and 10% were dead at 90 days (mRS 6). In contrast, patients treated after 6 h had a higher proportion of severe disability or death, with only 16.2% achieving functional independence (mRS 0–2). Patients in the 4.5-6-h group showed intermediate outcomes, with 28.8% achieving mRS 0–2. All mRS outcome frequencies stratified by treatment time are presented in Figure 1.

**Figure 2:**
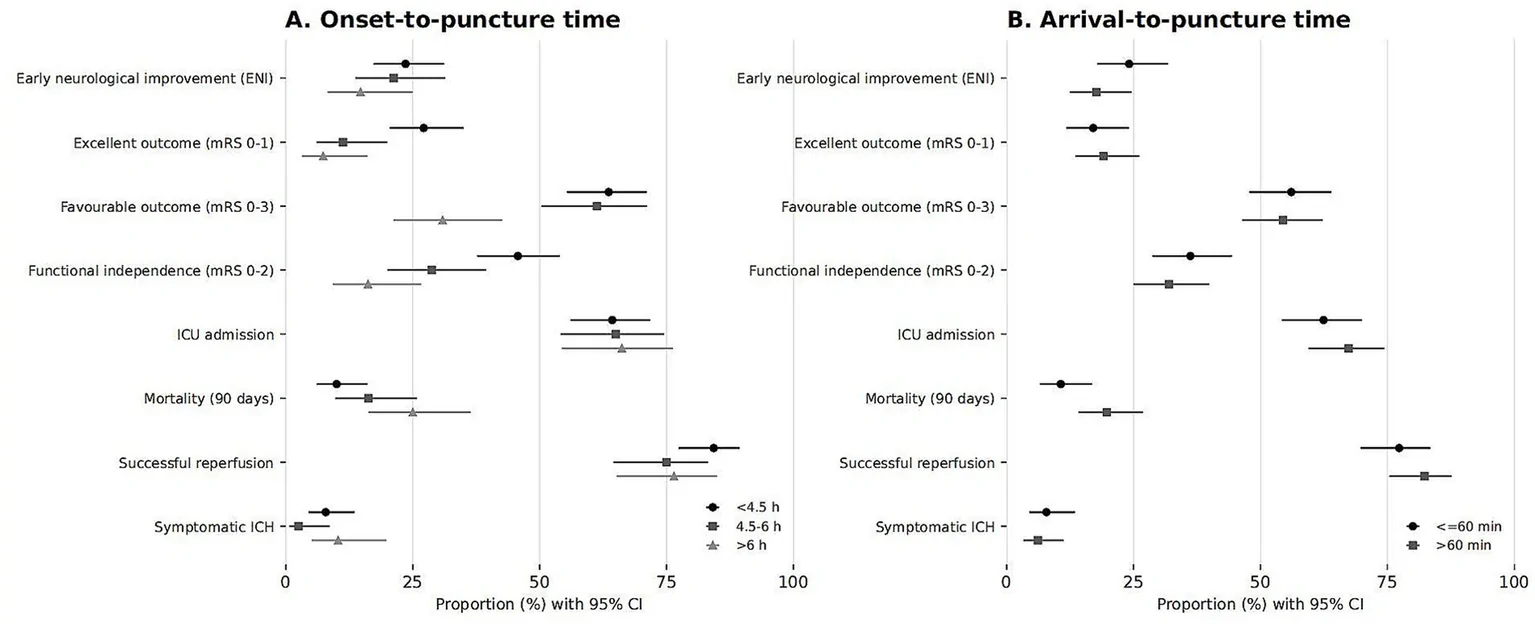
Forest plot of functional and safety outcomes by treatment time: **(A)** Functional and safety outcomes by onset-to-puncture time; **(B)** Functional and safety outcomes by arrival-to-puncture time.

A total of 187 patients (65%) required admission to the intensive care unit, while early neurological improvement was observed in 60 patients (21%). Symptomatic intracerebral haemorrhage within 36 h occurred in 20 patients (7%). Successful reperfusion was achieved in 230 patients (80%). Functional and safety outcomes varied across onset-to-puncture time windows (Figure 2A). Patients treated within 4.5 h demonstrated the most favourable results, with excellent outcome (mRS 0–1) in 27.1%, functional independence (mRS 0–2) in 45.7%, and favourable outcome (mRS 0–3) in 63.6%. Corresponding proportions were lower among patients treated between 4.5 and 6 h (11.3, 28.8, and 61.3%, respectively) and were lowest in those treated beyond 6 h (7.4, 16.2, and 30.9%). Early neurological improvement occurred in 23.6% of patients in the <4.5-h group, 21.3% in the 4.5-6-h group, and 14.7% in the >6-h group. Mortality increased with longer treatment delays, from 10.0% for onset-to-puncture <4.5 h to 16.3% for 4.5–6 h and 25.0% for >6 h. Rates of symptomatic intracerebral haemorrhage remained low across all groups (7.9, 2.5, and 10.3%, respectively). Successful reperfusion exceeded 70% in all time windows.

Similar patterns were observed when outcomes were stratified by arrival-to-puncture time (Figure 2B). Among patients treated within 60 min of hospital arrival, 17.0% achieved an excellent outcome, 36.2% achieved functional independence, and 56.0% achieved a favourable outcome. These proportions were slightly lower among patients with longer arrival-to-puncture times (19.0, 32.0, and 54.4%, respectively). Early neurological improvement was more frequent among patients treated within 60 min (24.1% vs. 17.7%), whereas mortality was lower (10.6% vs. 19.7%). Rates of symptomatic intracerebral haemorrhage remained similar (7.8% vs. 6.1%), and successful reperfusion rates were high in both groups (77.3% vs. 82.3%).

In adjusted analyses, longer onset-to-puncture time was associated with a graded reduction in the likelihood of favorable outcomes (Table 2). For each 1-h increase in onset-to-puncture time, the odds of a worse 90-day modified Rankin Scale (mRS) score increased by 8% (adjusted OR 1.08, 95% CI 1.02–1.14; *p* = 0.009). Similarly, each additional hour was associated with lower odds of achieving excellent functional outcome (mRS 0–1; adjusted OR 0.93, 95% CI 0.87–0.99; *p* = 0.028) and functional independence (mRS 0–2; adjusted OR 0.92, 95% CI 0.87–0.98; *p* = 0.011), as well as a 10% increase in the odds of 90-day mortality (adjusted OR 1.10, 95% CI 1.02–1.19; *p* = 0.018). Compared with patients treated within 4.5 h, those treated between 4.5 and 6 h showed no statistically significant shift toward worse functional outcome on the modified Rankin Scale (mRS) (adjusted odds ratio [OR], 1.08; 95% CI, 0.98–1.41; *p* = 0.11). In contrast, patients treated beyond 6 h demonstrated a significant shift toward worse functional outcomes (OR, 1.30; 95% CI, 1.12–1.62; *p* = 0.002). For dichotomized functional outcomes, treatment beyond 6 h was associated with lower odds of achieving excellent outcome (mRS 0–1; OR, 0.78; 95% CI, 0.64–0.95; *p* = 0.016) and functional independence (mRS 0–2; OR, 0.75; 95% CI, 0.58–0.96; *p* = 0.024), whereas the association with favorable outcome (mRS 0–3) did not reach statistical significance (OR, 0.83; 95% CI, 0.66–1.04; *p* = 0.10). There was no statistic significant association between onset-to-puncture time and successful reperfusion or symptomatic intracranial hemorrhage. Treatment beyond 6 h remained associated with higher 90-day mortality (OR, 1.33; 95% CI, 1.01–1.77; *p* = 0.045). Associations for patients treated between 4.5 and 6 h were smaller in magnitude and not statistically significant across dichotomized outcomes.

**Table 2:** Adjusted associations of onset-to-puncture time with clinical outcomes and complications

**Outcome**	**OR per 1-hour increase**	**P value**	**4.5–6 h vs <4.5 h**	**P value**	**>6 h vs <4.5 h**	**P value**
mRS (ordinal shift)	1.08 (1.02–1.14)	0.009	1.08 (0.98–1.41)	0.110	1.30 (1.12–1.62)	0.002
mRS 0–1	0.93 (0.87–0.99)	0.028	0.82 (0.56–1.20)	0.308	0.78 (0.64–0.95)	0.016
mRS 0–2	0.92 (0.87–0.98)	0.011	0.85 (0.60–1.22)	0.392	0.75 (0.58–0.96)	0.024
mRS 0–3	0.95 (0.89–1.01)	0.101	0.92 (0.67–1.25)	0.585	0.83 (0.66–1.04)	0.100
Mortality (90 d)	1.10 (1.02–1.19)	0.018	1.12 (0.74–1.69)	0.597	1.33 (1.01–1.77)	0.045
Successful reperfusion (eTICI ≥2b)	0.99 (0.94–1.05)	0.766	0.95 (0.61–1.49)	0.821	0.91 (0.57–1.44)	0.694
Symptomatic ICH	1.08 (0.95–1.23)	0.251	1.09 (0.52–2.28)	0.822	1.38 (0.67–2.82)	0.381

Median baseline neuron-specific enolase was 14.70 ng/mL, increasing to 17.60 ng/mL at 24 h, with a median change of 3.05 ng/mL. TNF-*α* and hsCR*p* values showed modest rises over the first 24 h (median changes 1.20 pg./mL and 0.70 mg/L, respectively). Measures of blood viscosity demonstrated small reductions, with whole-blood viscosity declining by −0.15 and plasma viscosity by −0.06. Fibrinogen levels remained largely stable, with a median change of −0.04 g/L. Overall, biomarker patterns reflected mild inflammatory activation and minimal haemorrheological variation during the early post-procedural period (Table 3).

**Table 3:** Baseline, 24-hour, and delta changes in biomarkers following endovascular thrombectomy.

Biomarker	Baseline median (IQR)	24-hour median (IQR)	Change median (IQR)
NSE (ng/mL)	14.70 (11.30–17.52)	17.60 (13.78–22.72)	3.05 (−2.52–9.02)
TNF-α (pg/mL)	10.20 (8.00–12.10)	11.10 (8.28–14.10)	1.20 (−2.60–4.53)
hsCRP (mg/L)	6.90 (4.77–8.90)	8.00 (5.07–10.62)	0.70 (−2.23–5.03)
Whole-blood viscosity (mPa·s)	4.00 (3.62–4.45)	3.88 (3.45–4.28)	−0.15 (−0.67–0.39)
Plasma viscosity (mPa·s)	1.49 (1.35–1.63)	1.44 (1.32–1.57)	−0.06 (−0.23–0.11)
Fibrinogen (g/L)	3.08 (2.62–3.63)	3.05 (2.64–3.59)	−0.04 (−0.74–0.63)

Fugl-Meyer motor recovery at 90 days showed broadly comparable scores across the onset-to-puncture groups (Table 4), with median values of 57.3 (IQR 49.2–67.9) for patients treated within 4.5 h, 56.8 (IQR 51.1–64.3) for those treated between 4.5 and 6 h, and 58.8 (IQR 43.5–65.2) among patients treated beyond 6 h. In contrast, more pronounced differences were observed when stratifying by in-hospital workflow times. Patients who achieved arterial puncture within 60 min of hospital arrival had substantially higher Fugl-Meyer scores, with a median of 61.9 (IQR 52.3–71.0), compared with 54.6 (IQR 44.7–62.8) among those with door-to-puncture times longer than 60 min.

**Table 4:** Fugl-Meyer motor scores at 90 days by treatment time categories.

Timing category	*n*	Median (IQR)
Onset-to-puncture time		
<4.5 h	140	57.3 (49.2–67.9)
4.5–6 h	80	56.8 (51.1–64.3)
>6 h	68	58.8 (43.5–65.2)
Arrival-to-puncture time		
≤60 min	141	61.9 (52.3–71.0)
>60 min	147	54.6 (44.7–62.8)

## Discussion

In this study, we evaluated the association between treatment timing and clinical outcomes in patients with acute ischemic stroke undergoing endovascular therapy. Shorter onset-to-puncture times were associated with more favorable functional outcomes at 90 days; however, the magnitude of these associations was modest after adjustment and varied across outcome measures. Patients treated within 4.5 h of onset had the highest proportions of excellent, independent, and favorable functional outcomes, whereas those treated beyond 6 h demonstrated progressively lower rates of recovery and a higher risk of death. After accounting for baseline clinical severity and occlusion characteristics, these associations were attenuated, indicating that anatomical and biological heterogeneity partially explains outcome differences across treatment-time windows. Nevertheless, the overall directional consistency across analyses suggests that treatment delay remains an important contextual marker of recovery potential in routine practice.

The distribution of outcomes across treatment-time categories in our cohort aligns closely with the well-established time dependency of EVT benefit. The HERMES meta-analysis (5) demonstrated that faster reperfusion translated into a higher likelihood of functional independence, with every hour of delay reducing the odds of a favorable outcome by approximately 5%. Similar patterns have been documented in large observational registries, including the MR CLEAN Registry (15, 18), where treatment delays were strongly associated with reduced functional independence and increased mortality. Our findings extend this body of evidence by illustrating how these time–outcome relationships manifest in a real-world EVT population shaped by patient selection and anatomical variability, rather than replicating trial-derived effect estimates. Although the effect sizes observed in our analysis were smaller than those reported in highly selected trial populations, the attenuation of associations after adjustment highlights the contribution of occlusion anatomy and baseline stroke severity to observed outcome gradients, while preserving the overall directionality of the time–outcome relationship.

The pattern observed for early neurological improvement supports this interpretation. ENI was two to three times more common among patients treated earlier, reflecting the capacity of hyperacute reperfusion to rescue ischemic penumbra before irreversible infarction occurs. This is consistent with perfusion imaging studies demonstrating that the volume of salvageable tissue declines rapidly in the first hours after onset, particularly in patients with poor collateral circulation. Such patients may experience accelerated infarct growth beyond 6 h, which likely contributes to the lower probability of achieving functional independence in the >6-h group.

Safety outcomes also aligned with established evidence. Rates of symptomatic intracranial hemorrhage were low across all time categories, consistent with reported rates of 4 to 8% in previous studies (19, 20). We did not observe an increase in hemorrhagic complications among patients treated before or after 6 h, supporting existing evidence that EVT does not substantially increase hemorrhage risk even in extended windows when appropriate patient selection is applied. Similarly, reperfusion success rates exceeded 75% in each time category, comparable to contemporary multicenter registries that report eTICI ≥2b reperfusion rates between 70 and 90% (21, 22). The stability of reperfusion performance across time windows indicates that differences in clinical outcomes are unlikely to be attributable to procedural difficulty or operator performance, but rather to the diminishing biological opportunity for tissue salvage as time progresses.

Mortality at 90 days showed a clear gradient, with the highest mortality among patients treated beyond 6 h. This pattern mirrors findings from both clinical trials and real-world cohorts. In the MR CLEAN Registry, adjusted mortality increased significantly with each hour of delay in reperfusion. Similar results have been reported in previous studies (23, 24), where patients treated later or with greater infarct burden had substantially higher mortality. In our study, the >6-h group had lower rates of independent and favorable outcomes and a higher burden of severe disability, which likely contributed to the observed mortality pattern. The association between later treatment and mortality persisted even after adjustment, suggesting that time to treatment, independent of baseline risk factors, remains an important determinant of survival.

Our findings reinforce the central message that has emerged across EVT research: although modern workflows have improved speed and technical success, the therapeutic benefit of EVT remains highly time dependent. Even within a cohort with relatively efficient treatment times and high reperfusion rates, earlier treatment was consistently associated with improved functional outcomes, more frequent neurological recovery, and lower mortality, without evidence of increased hemorrhagic risk. These findings emphasize the continued need for system-level interventions (25) to reduce treatment delays, including streamlined in-hospital processes, rapid imaging-to-puncture workflows, and optimized transfer pathways for patients initially presenting to non-thrombectomy centers.

This study has several limitations. It was conducted within a single endovascular centre, which limits the ability to generalise findings to broader health systems with different prehospital pathways, imaging protocols, staffing structures, and procedural experience. Treatment times in high-volume centres are often shorter than those reported in routine clinical care across many regions, and workflow performance is known to vary substantially between hospitals. As a result, the magnitude of benefit associated with earlier treatment in this analysis may differ in settings with longer system delays or less structured stroke pathways. The sample size limited the precision of estimates for secondary and safety outcomes, particularly symptomatic intracranial haemorrhage and mortality. These endpoints had relatively small event counts and the confidence intervals remain wide. Time intervals were derived from clinical records and self-reported, and onset time in particular may be imprecise for unwitnessed strokes, which could introduce exposure misclassification. Imaging selection criteria were not standardised and collateral status, infarct progression rate, and perfusion-defined salvageable tissue were not available. These factors are known to modify the relationship between time and treatment effect and could not be incorporated into the adjusted models.

Future work should widely validate these findings in larger multicentre cohorts that include diverse hospital systems and more granular workflow data. Integration of perfusion imaging, collateral grading, and advanced physiological markers would allow clearer assessment of how treatment delays influence infarct growth and reperfusion injury. Prospective evaluations of targeted workflow interventions, including prehospital large vessel occlusion identification, streamlined imaging protocols, and direct-to-angiography approaches, are needed to determine which strategies most effectively reduce time to reperfusion. Future studies would also benefit from incorporating patient-reported outcomes and cognitive measures to characterise the broader recovery trajectory after endovascular therapy.

## Conclusion

In this cohort of patients with acute ischemic stroke due to large-vessel occlusion treated with endovascular thrombectomy, shorter onset-to-puncture times were associated with more favorable functional outcomes at 90 days, whereas treatment beyond 6 h was associated with worse functional status and higher mortality. These associations were attenuated after accounting for clinical severity and occlusion characteristics, indicating that anatomical and biological heterogeneity partially explains outcome differences across treatment-time windows. Treatment timing was not meaningfully associated with successful reperfusion or symptomatic intracranial hemorrhage. Overall, these findings underscore the importance of minimizing avoidable delays in acute stroke care while emphasizing that time-based metrics alone incompletely capture biological treatment opportunity in contemporary practice.

## Data Availability

The original contributions presented in the study are included in the article/supplementary material, further inquiries can be directed to the corresponding author.
